# HDL Receptor in *Schistosoma japonicum* Mediating Egg Embryonation: Potential Molecular Basis for High Prevalence of Cholesteryl Ester Transfer Protein Deficiency in East Asia

**DOI:** 10.3389/fcell.2022.807289

**Published:** 2022-03-17

**Authors:** Shinji Yokoyama

**Affiliations:** Food and Nutritional Sciences, Chubu University, Kasugai, Japan

**Keywords:** *Schistosoma japonicum*, HDL, egg embryonation, CD36, cholesteryl ester, CETP, East Asia, hepatic granulomatosis

## Abstract

Schistosomiasis is a life-threatening parasitic disease caused by blood flukes, Schistosomes. In its intestinal type, the parasites reside in visceral/portal veins of the human hosts and lay eggs to excrete in feces *via* intestinal tracts, and some of the aberrant eggs plug into the liver *via* the portal blood flow. Ectopic growth of these eggs causes fatal granulomatosis and cirrhosis of the liver. The parasites ingest nutrients from the host blood plasma by using nonspecific and specific transport *via* their body surface and alimentary tracts. It is especially important for the female adults to obtain lipid molecules because they synthesize neither fatty acids nor sterols and yet produce egg yolk. Low-density lipoprotein receptors have been identified in the body of the Schistosomes but their functions in the parasite life cycle have not clearly been characterized. On the other hand, CD36-related protein was identified in the body and the eggs of Asian blood fluke, *Schistosoma japonicum*, and characterized as a molecule that mediates selective uptake of cholesteryl ester from the host plasma high-density lipoproteins (HDLs). This reaction was shown crucial for their eggs to grow to miracidia. Interestingly, abnormal large HDL generated in lack of cholesteryl ester transfer protein (CETP) is a poor substrate for this reaction, and, therefore, CETP deficiency resists pathogenic ectopic growth of the aberrant parasite eggs in the liver. This genetic mutation is exclusively found in East Asia, overlapping with the current and historic regions of *Schistosoma japonicum* epidemic, so that this infection could be related to high prevalence of CETP deficiency in East Asia.

## Introduction: Schistosomiasis

Infection of blood flukes, schistosomiasis, is a life-threatening parasitosis widely spread in the world, caused by species such as *Schistosoma mansoni* (*S. mansoni*) in Africa and South America, *Schistosoma japonicum* (*S. japonicum*) or its subtypes in East Asia (Gryseels et al., 2006; McManus et al., 2018), and *Schistosoma haematobium* (*S. haematobium*) in Africa and Middle East. The former two cause intestinal schistosomiasis where the adult parasites reside in portal/visceral veins of the host animals and lay eggs which penetrate into intestinal lumen to be excreted in feces. The latter causes urogenital schistosomiasis where the worms infect in pelvic vein and the eggs penetrate into urinary tract and genital organs to be excreted (McManus et al., 2018). The eggs grow to miracidia by using their yolk and hatch in environmental fresh water. They reach the intermediate hosts, specific fresh water snails such as *Oncomelenia nosophora*, where they grow to sporocysts and then to cercariae (McManus et al., 2018). The cercariae go into the water again and infect the final mammalian host by penetrating their skins and migrating into their blood stream to reach the final parasitic position. Thus, endemic of schistosomes seems associated with extensive exposure to natural fresh water reserve of the hosts in everyday life. *S. japonicum* has been endemic widespread in East Asia such as Indonesia, Philippines, China, and Japan as far as traced in recorded history. Its Mekong strain or *S. mekongi* is also found in the Mekong basin in Indochina peninsula. *S. japonicum* was first identified in Japan early 20th century when some intensively infected regions were still apparent (Iida et al., 1999). It used to be one of the major infectious diseases among rice farmers in certain areas of Japan but has been eradicated by eliminating the intermediate hosts of fresh water snails by transforming irrigation channels for rice fields into concrete (Iida et al., 1999; Ohmae et al., 2003). The similar environmental strategy has been adopted in China, where the number of the infected patients has sharply come down in the past decades from 11 million to several hundred thousand (Wang et al., 2009; Qian et al., 2018), but it still remains as one of the major public health problems there (Wang et al., 2018), especially in such occasions as flooding (McManus et al., 2010; Yang et al., 2018; Xue et al., 2021). *S. japonicum* is still active in Philippines (Bergquist and Tanner, 2010; Gordon et al., 2012), Cambodia, Laos, Thailand, Malaysia, and Indonesia (Harinasuta, 1984) accounting for 2 million patients, and it remains as the second major intestinal schistosomiasis next to the African blood fluke, *S. mansoni*.

One of the major fatal complications of intestinal schistosomiasis is the cirrhosis of the liver in the intestinal type. Although the eggs laid are to be excreted and get into the normal life cycle, substantial portion of them aberrantly reaches and plugs into the liver by the upward flow of the portal blood and ectopically grow to miracidia there (Cheever et al., 1994). This process causes granulomatosis lesions and eventually fatal cirrhosis in the liver. In the urogenital type, the eggs cause granulomatosis in urinary tract or genital organs. Therefore, the egg embryonation is a potential target for prevention of fatal development of the schistosomiasis although the mechanism to induce this lesion is still unknown. Specific antibodies against the various egg antigens have been identified as markers of the infection, but their relationship to granulomatogenesis is unclear (Mohda et al., 1998). A potential pathogenesis stage is egg embryonation to miracidium, because the eggs only after this stage cause the hepatic lesion when transplanted (Hirata et al., 1991). Host L-selectin binds to the eggs only at the stage of miracidium (El Ridi et al., 1996). Accordingly, vaccination to stabilize the embryonation has been proposed as an anti-schistosomiasis therapy (Mitchell et al., 1994).

Schistosomes ingest nutrients from the host blood by nonspecific and specific absorption *via* the body surface tegument and alimentary tract (Skelly et al., 2014). It is especially important for them to gain lipid nutrients because the parasites *de novo* synthesize neither fatty acid nor sterol molecules (Smith et al., 1970; Brouwers et al., 1997), and more so in female as they need to produce the yolk for the eggs they lay. As these parasites reside in blood stream, it is rational to assume that the major sources of the lipid nutrients are the host plasma lipoproteins. Mammalian plasma lipoproteins are largely classified to those containing apolipoprotein B (apoB), such as low-density lipoproteins (LDLs), very low-density lipoproteins (VLDLs), and high-density lipoproteins (HDLs). ApoB lipoproteins mainly function to deliver fatty acids and cholesterol from the liver to the tissues and HDL recovers cholesterol from the tissues to transport back to the liver for catabolic conversion to bile acids. The former are taken up by the endocytotic LDL receptor pathway, whereas cholesteryl ester (CE) molecules of HDL are selectively taken up by the CD36-like molecules such as scavenger receptor B1 (SR-B1) in rodents (Acton et al., 1996) or LIMPIIanalogous-1 (CLA-1) in humans (Imachi et al., 1999a; Imachi et al., 1999b). The LDL receptor–like molecules have been identified both in *S. mansoni* (Rumjanek et al., 1983; Rumjanek et al., 1985; Rumjanek et al., 1988; Bennett and Caulfield, 1991; Xu and Caulfield, 1992; Furlong et al., 1995; Tempone et al., 1997) and *S. japonicum* (Rogers et al., 1989; Rogers et al., 1990; Fan et al., 2003), and CD36-like molecules have also been identified in *S. mansoni* (Dinguirard and Yoshino, 2006) and *S. japonicum* (Okumura-Noji et al., 2013; Zhang et al., 2013). The latter was shown critical for the eggs to grow miracidia (Okumura-Noji et al., 2013) and abnormal large HDL particles generated in patients with CE transfer protein (CETP) deficiency were shown to be a poor substrate for this reaction (Okumura-Noji et al., 2001). The gene mutations to cause this disorder are exclusively found in East Asia, potentially being associated with the historical endemic range of *S. japonicum* (Yokoyama, 2014; Yokoyama, 2015; Yokoyama et al., 2015).

## LDL Receptor–Like Molecules in Schistosomes

LDL is one of the major lipid protein complexes in blood plasma and thought to transport lipid molecules from the liver to the peripheral tissues *via* circulating blood plasma. Cells take up LDL particles by the endocytotic LDL receptor to transport them by endosome to lysosome, where the lipid and protein molecules are hydrolyzed and cholesterol molecules are then intracellularly redistributed (Brown and Goldstein, 1986). The LDL receptor was defined as the major catabolic pathway to clear plasma LDL by identifying that its functional deficiency is the causative factor of familial hypercholesterolemia (Brown and Goldstein, 1974a). This pathway is the first well-characterized reactions at cellular and molecular biology levels in detail for trafficking and metabolic regulation of cholesterol (Brown and Goldstein, 1974b). As LDL is the major lipoprotein in human plasma and the LDL receptor is the best characterized molecules for the uptake of extracellular cholesterol, it is naturally conceivable that this most abundant lipid carrier would be a major source of lipid nutrients for blood flukes as well. LDL-specific binding components were, in fact, identified in the tegument of *S. mansoni* cercariae as a protein of 45 kDa(Rumjanek et al., 1983; Rumjanek et al., 1985; Rumjanek et al., 1988), much smaller than the mammalian LDL receptor of 95 kDa (Yamamoto et al., 1984), being induced by incubation with human serum. Other reports demonstrated the proteins with even smaller molecular weights of some 15.7 and 17.8 kDa (Xu and Caulfield, 1992) or 14, 35, and 60 kDa (Tempone et al., 1997) to interact with human LDL in a specific manner. A similar molecule of 43 kDa was identified in the *S. japonicum* tegument for specific interaction with human LDL (Rogers et al., 1989; Rogers et al., 1990). Ingestion and subsequent intracorporeal distribution of LDL were also demonstrated in *S. mansoni* by using LDL containing fluorescence-labeled hydrophobic molecules of 3,3′-diindolylmethene (DiI) (Bennett and Caulfield, 1991) or boron-dipyrromethene– and nitrobenzoxadiazole-coupled phosphatidylcholines (Furlong et al., 1995). Another molecule was identified in the Asian blood fluke *S. japonicum* from its cDNA library deduced to the sequence of 207–amino acid residues containing a transmembrane domain in the C-terminal and a Cys-rich motif in the N-terminal homologous to the Cys-rich repeats of the ligand binding domains in the mammalian LDL/VLDL receptors (Fan et al., 2003). However, recent proteomics analysis of surface proteins of *S. mansoni* failed to prove the presence of any homologous protein to the mammalian LDL receptors (Braschi et al., 2006a; Braschi et al., 2006b; Braschi and Wilson, 2006). No functional analysis has been successful to demonstrate physiological relevance of any of these LDL receptor–like molecules for a major ingesting mechanism of the lipid nutrients for schistosomes (Skelly et al., 2014).

## HDL as a Potential Functional Source of Lipid Nutrient for Schistosomes

HDL is another major lipid-protein particle in human blood plasma to carry lipids. HDL is thought to transport lipid molecules from the somatic cells to the liver, especially cholesterol molecules as a part of their catabolic pathway. Cholesterol molecule is not catabolized in most of somatic cells in animals so that it must be exported from the cells and transported to the liver where it is converted to bile acids for excretion. However, cellular uptake of the lipid molecules carried by HDL is much less characterized than that of LDL. It has been implicated that lipid uptake pathway by cells is different between LDL and HDL. Unlike endocytotic uptake of LDL particles by the LDL receptor and subsequent lysosomal hydrolysis of its lipid molecules (Brown and Goldstein, 1986), cells have been shown to take up selectively CE from HDL particles (Rinninger and Pittman, 1987; Goldberg et al., 1991; Green and Pittman, 1991; Khoo et al., 1995). CE derived from HDL was shown hydrolyzed even in the fibroblasts of patients with Wolman’s disease where LDL-CE is not hydrolyzed due to deficiency of lysosomal acid lipase (Sparrow and Pittman, 1990). SR-B1, a membrane protein closely related to CD36 widely functional in cellular immune response, has been identified as a mediator for selective uptake of CE from HDL particle in mouse (Acton et al., 1996). This reaction was demonstrated crucial for the mouse adrenal glands to generate glucocorticoid *in vivo* (Hoekstra et al., 2012). CLA-1 as a human counterpart of SR-B1 was also shown to function in cellular CE uptake from HDL (Imachi et al., 1999a; Imachi et al., 1999b; Hoekstra et al., 2012). However, it is puzzling that large increase is found in unesterified cholesterol but not CE in the HDL fraction of SR-B1–deficient mice (Hildebrand et al., 2010). More recently, Aster proteins were found to facilitate transfer of HDL-derived cholesterol from plasma membrane to endoplasmic reticulum, but no information is provided for CE (Sandhu et al., 2018). Thus, study of this pathway is still preliminary, and the mechanisms are largely unknown for selective CE uptake, its intracellular trafficking and its extralysosomal hydrolysis (Connolly et al., 1996; Pollard et al., 2015; Gillard et al., 2017; Shen et al., 2018). To make it more confusing, scavenger receptors have been proposed to play a key role in atherogenesis by unregulated uptake of modified LDL such as oxidized LDL generated by retention of LDL in blood plasma (Dhaliwal and Steinbrecher, 1999; Van Berkel et al., 2000; Greaves and Gordon, 2009).

**FIGURE 1:**
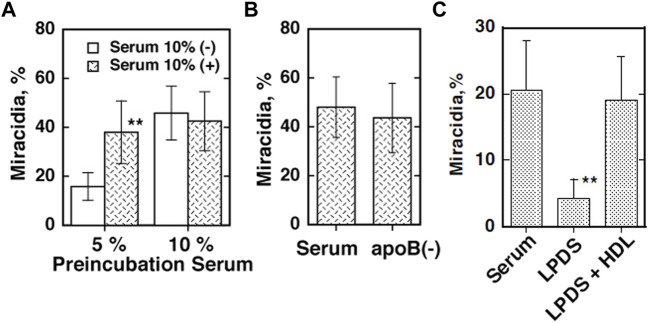
Embryonation/maturation of the *S. japonicum* eggs in culture (Okumura-Noji et al., 2013). The percentage of miracidia in the total eggs per one pair of the adult parasites cultured for 10 days was counted and estimated as efficiency of embryonation. **(A)** The eggs were separated from the adults after 2 days of culture in the medium containing five or 10% human serum and cultured for 8 days in the fresh media with or without 10% serum. **(B)** The eggs were removed from the parents cultured in the same condition as A with 5% serum and further cultured in 10% of whole serum and its *d* = 1.063 bottom fraction [apoB (−)]. **(C)** A pair of the parent adults was cultured for 10 days in the media with lipoprotein-depleted serum (LPDS) (4 mg protein/ml) with or without isolated HDL fraction (150 μg cholesterol/ml). The numbers of the adult pairs assayed were 6 **(A)** and 8 **(B,C)**. The data represented the average and SE. **p < 0.005 and *p < 0.05.

**FIGURE 2:**
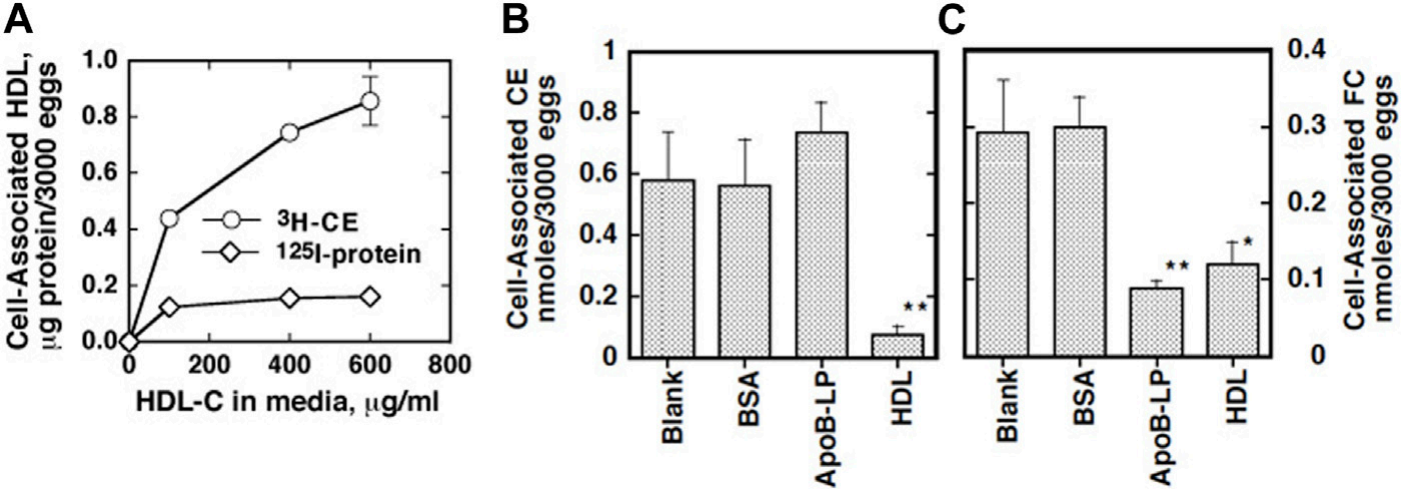
Specific cholesterol uptake from lipoproteins by *S. japonicum* eggs (Okumura-Noji et al., 2013). **(A)** Human HDL was labeled with [^3^H]CE or ^125^iodine. The *S. japonicum* eggs were incubated with [^3^H]CE-HDL or ^125^I-HDL at 37 and 4°C for 20 h. Association of HDL the eggs was estimated as HDL protein calculated from each specific activity. Specific association was estimated by displacement by 10 times of the amount of non-labeled HDL and difference between the results at 37°C and 4°C. **(B and C)** Selective uptake of cholesterol by *S. japonicum* eggs from HDL. HDL was double-labeled with [^3^H]CE and [^14^C]cholesterol and incubated with the *S. japonicum* eggs in the presence of 10 times of excess of non-labeled HDL, non-labeled apoB lipoprotein (ApoB-Lp) or BSA (1 μg/ml). Uptakes of CE **(B)** or cholesterol [FC, **(C)**] were calculated as the difference between the specific values at 37°C and 4°C. The data represent the average and SE of the triplicate assay. **p < 0.01 and *p < 0.05 different from “Blank”.

The eggs of *S. japonicum* undergo growth to miracidia by using their yolk after they are laid and excreted and hatch in fresh water in the environment (Skelly et al., 2014). This embryonation process to miracidia was found to require the presence of plasma HDL but not apoB lipoproteins (LDL and VLDL) in direct culture, and pre-exposure of the parents to HDL partially rescues their eggs to grow (Figure 1) (Okumura-Noji et al., 2013). In addition, the eggs were shown to take up selectively CE from HDL but not from LDL (Figure 2) (Okumura-Noji et al., 2013). The nature of the reaction is consistent with CE uptake by SR-B1 (Acton et al., 1996). Thereby, HDL was implicated to be more important nutrients source than LDL for generating the egg yolk of *S. japonicum*.

**FIGURE 3:**
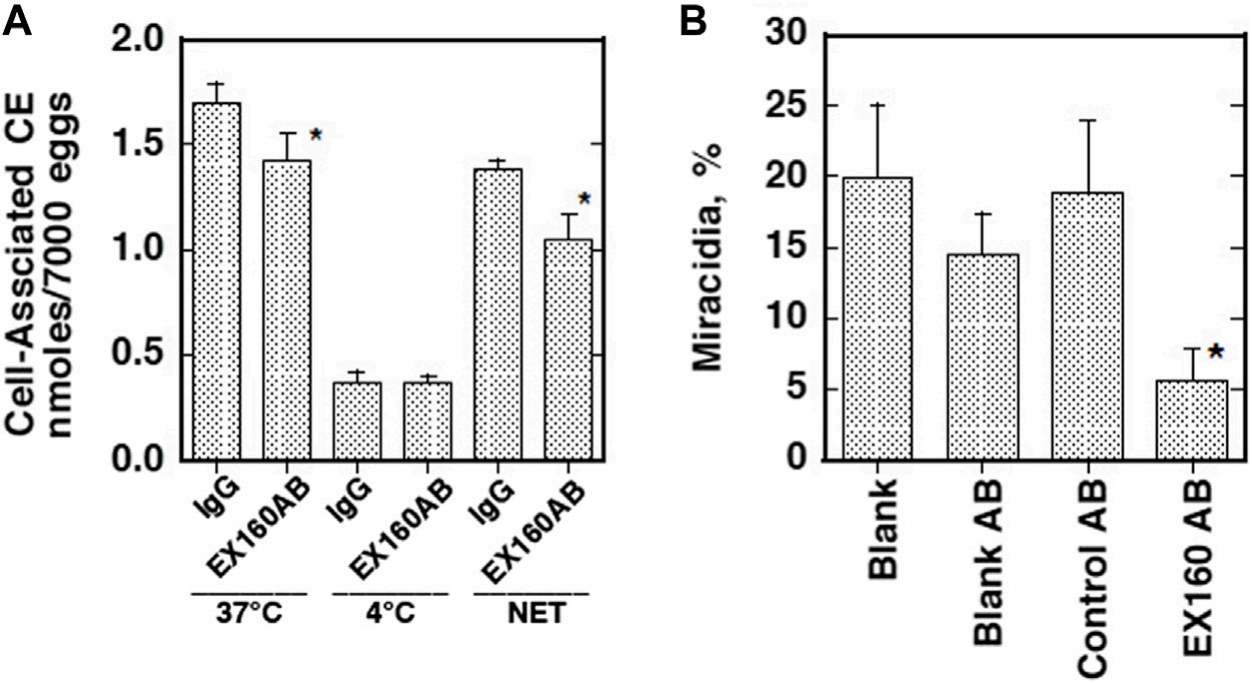
Suppression of HDL-CE uptake and maturation of the eggs by the antibody against extracellular loop of CD36RP (EX160AB) (Okumura-Noji et al., 2013). **(A)** Uptake of HDL-CE was measured in the same system as used in Figure 2, in the presence of the antibody. **(B)** Maturation of *S. japonicum* eggs was estimated in the same condition as Figure 1 except for using 5% serum, in the presence of the antibodies. IgG, non-immune rabbit IgG; EX160AB, the antibody against Ex160. Blank, with no additional antibody/antisera. Blank AB, with 1/100 volume of non-immune rabbit serum; Control AB, with 1/100 volume of rabbit antisera against the intracellular domain peptide of CD36RP (anti-peptide 331–348, anti-P); the titer of Ex160 was adjusted to less than 1/25 of the anti-P antisera. Data represent M ± SE of *n* = 6 for each group. An asterisk indicates *p* < 0.05 against IgG, Blank, Blank AB, and Control AB.

**FIGURE 4:**
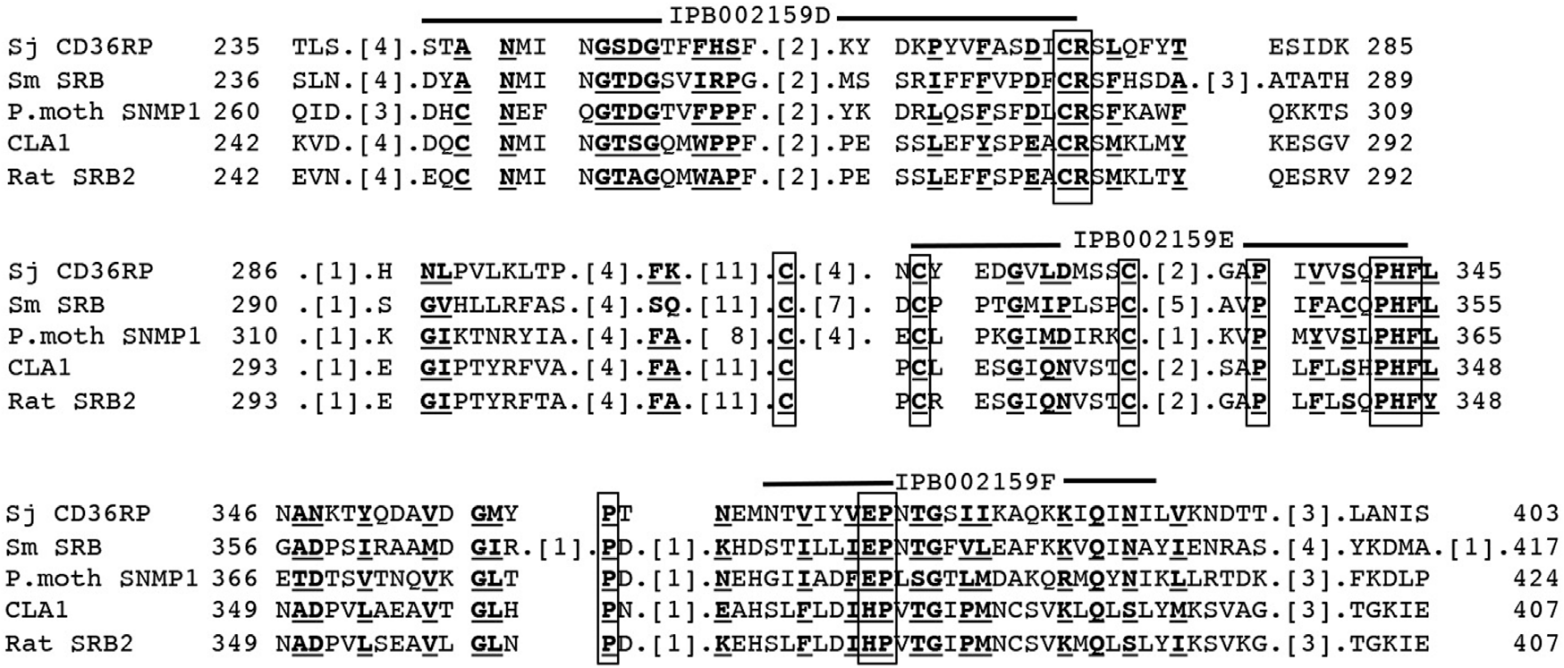
Multi-alignment of the amino acid sequences of CD36RP of *S. Japonicum* (Sj CD36RP) (Okumura-Noji et al., 2013) in comparison to other CD36 family proteins, *S. mansoni* scavenger receptor B (CD36-like protein) (Sm SRB) (Dinguirard and Yoshino, 2006), sensory neuron membrane protein of *Polyphemus* moth (P.moth SNMP-1), CLA1 as a human counterpart of SR-B1, and rat SR-B1. The amino acid residues in bald letters and underlined letters are conserved in 32 proteins from a variety of organisms. Conserved Cys and Pro residues in CD36 blocks IPB002159D to F regions are indicated as boxed.

CD36-like class B scavenger receptor proteins were identified in schistosomes. Dinguirard and Yoshino reported a CD36-like protein in *S. mansoni* and demonstrated its binding to acetylated LDL in the sporocytes (Dinguirard and Yoshino, 2006). However, it has never been characterized for either the interaction with HDL or any physiological function. The presence of a similar molecule was demonstrated as a tegument-exposed protein in both male and female adults of *S. japonicum* (Zhang et al., 2013). This CD36-related protein (CD36RP) has been fully identified in *S. japonicum* by cloning from its cDNA library with 1,880 bp encoding 506–amino acid residues, including the CD36 domains and two transmembrane regions, and expressed in the adults and the eggs (Okumura-Noji et al., 2013). This protein was demonstrated to interact selectively with HDL by its extracellular loop domain, and the antibody against this region was shown to inhibit selective CE uptake from HDL and embryonation to miracidia of the eggs of *S. japonicum* (Figure 3) (Okumura-Noji et al., 2013). Thus, this protein likely mediates CE uptake by the parasites from HDL as a key reaction for growth and embryonation to miracidia of the eggs of *S. japonicum*. The two proteins from *S. mansoni* and *S. japonicum* are homologous including highly conserved four Cys and four Pro residues in the domains from IPB002159D to IPB002159F like other CD36 family proteins (Figure 4) (Okumura-Noji et al., 2013), so that CD36-like protein in *S. mansoni* may also function to interact with HDL.

HDL has been claimed to have “beneficial” effects other than cholesterol transport, such as antioxidative or anti-inflammatory functions, which may be mediated by sphingosine-1-phosphate concentrated on HDL to induce intracellular signaling pathway (Nagao et al., 2018). However, these reactions have not been claimed for association with CD36-type HDL receptor.

**FIGURE 5:**
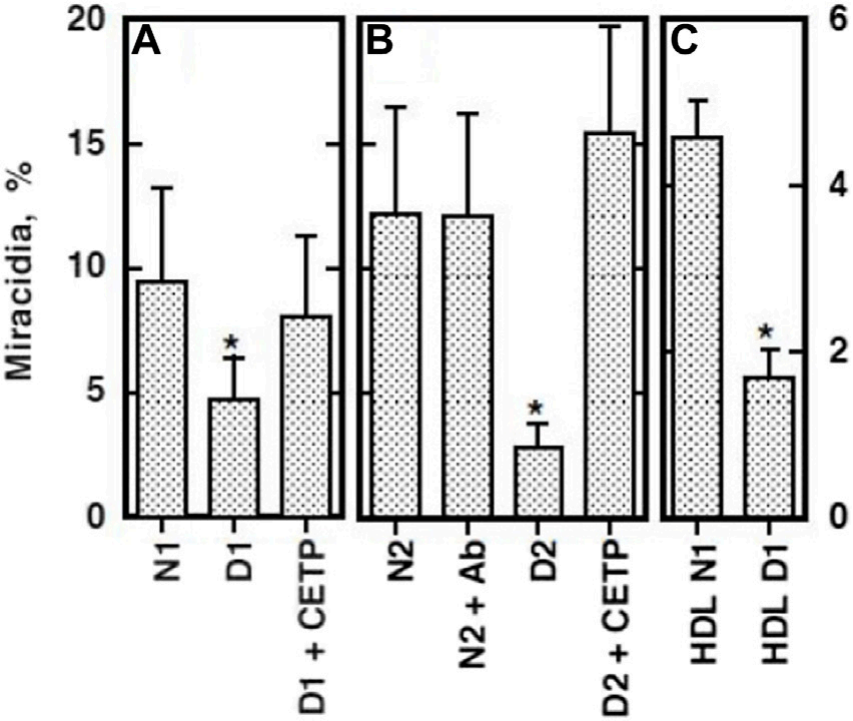
Embryonation of the eggs of *S. japonicum in vitro* in the presence of HDL from patients with CETP deficiency (Okumura-Noji et al., 2001). Egg embryonation was estimated when an adult pair of the parasite was cultured for 8 and 10 days **(A–C)**, respectively). The culture media contained 10% normal human sera (N1 and N2) and CETP-deficient human sera (D1 and D2). Patients with CETP deficiency were both identified as intron 14 splicing defect. Ab indicates the presence of anti-CETP inhibitory monoclonal antibody (IgG). CETP indicates presence of purified human CETP. HDL N1 and HDL D1 indicate the HDL isolated from normal human plasma (N1) or the CETP-deficient plasma (D1). Each panel represents an independent experiment, in which the data points are the average ± SE of the four **(A, C)** and eight **(B)** measurements. *p < 0.05 different from N. *p = 0.059 between HDL N1 and HDL D1.

**FIGURE 6:**
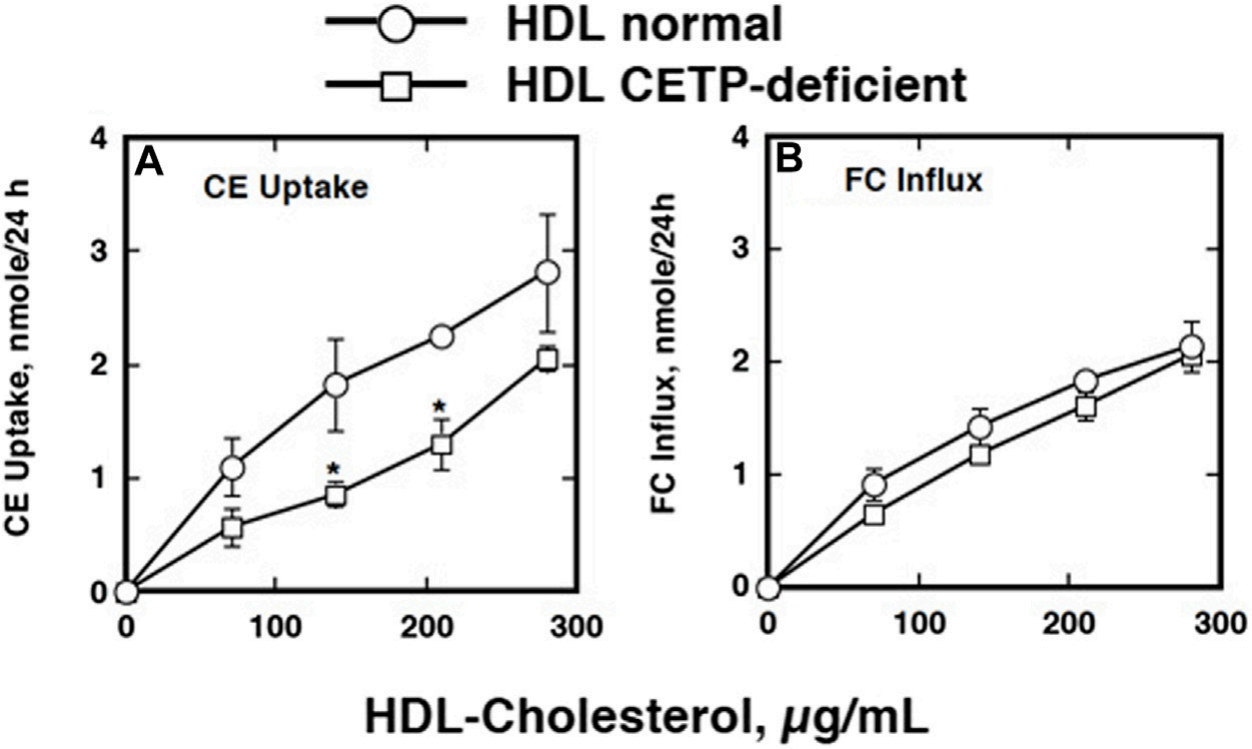
Active CE uptake by the eggs of *S. japonicum* from normal HDL and that isolated from CETP deficiency (Okumura-Noji et al., 2001). HDL was labeled with [^3^H]CE and active uptake was determined by the difference between the values at 37°C and 4°C. The data represent the average and SE of the triplicate assay. Symbols are as follows: circles, HDL prepared from normal human plasma; squares, CETP-deficient human plasma. *p < 0.05 between normal HDL and CETP-deficient HDL. Panel **(A)**, CE uptake. Panel **(B)**, FC influx.

**FIGURE 7:**
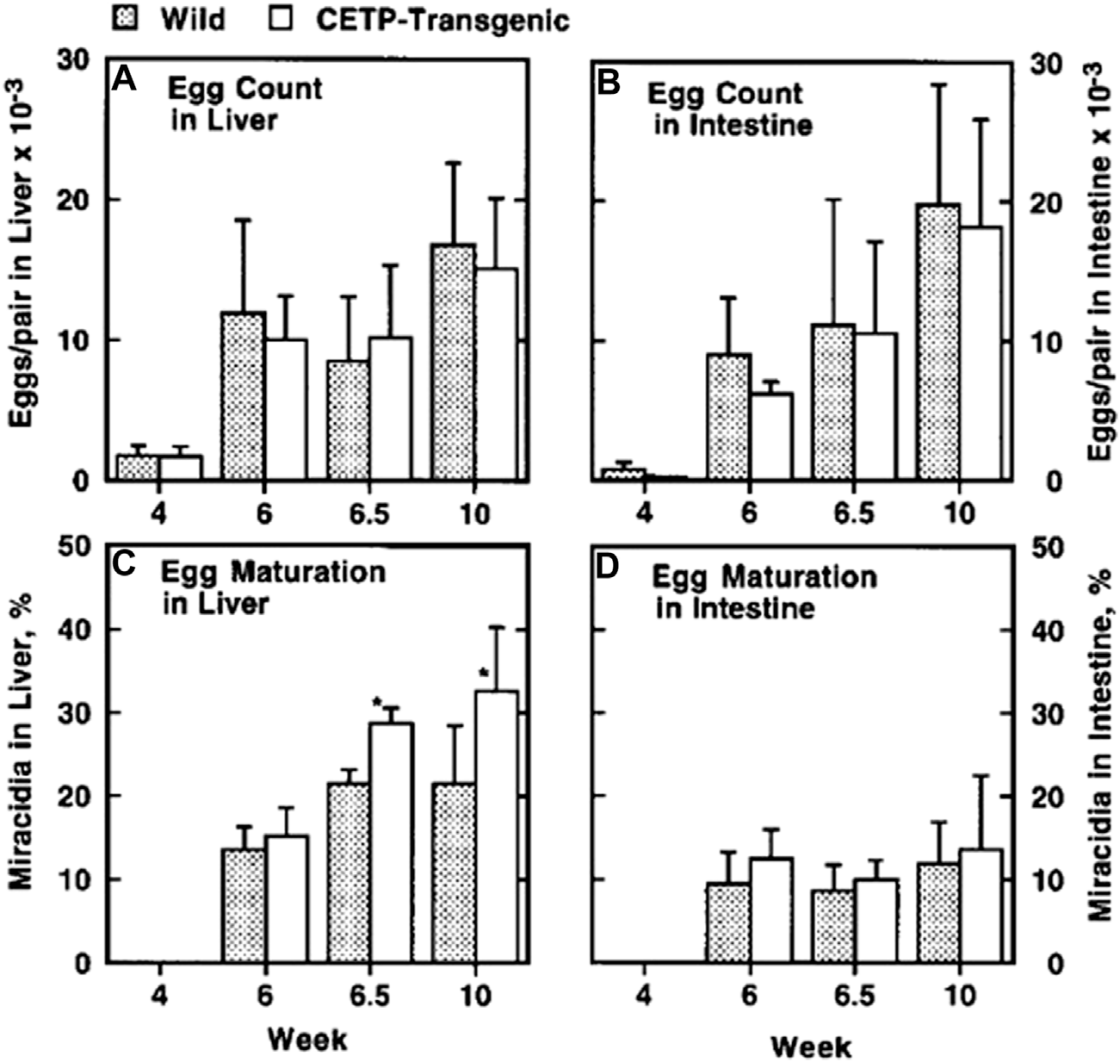
Egg analysis in the mice infected by *S. japonicum* (Okumura-Noji et al., 2001). The CETP transgenic and wild-type mice were infected. Total eggs recovered were counted in the liver and intestine and the egg number per an adult pair of the parasite in the portal vein was calculated for individual mouse. Egg embryonation to miracidia was microscopically counted for 100 eggs, for the liver and intestine in each mouse. **(A,B)** The egg count, in the liver and in the intestine. No difference was observed between wild type C57BL/6 mice and human CETP-transgenic mice. **(C,D)** The egg embryonation as indicated by the relative content of miracidia. In the CETP-transgenic mice, the eggs were matured significantly more in the liver by *p < 0.001, whereas there is no difference in the intestine. *N* = 10.

The findings above were supportive to and providing molecular basis for the preceding interesting observations about the function of HDL from the patients CETP deficiency for embryonation of *S. japonicum* eggs. The eggs of *S. japonicum* require HDL to embryonate to miracidia as mentioned above. Growth of the *S. japonicum* eggs to miracidia was retarded in the plasma of patients with CETP deficiency (Okumura-Noji et al., 2001). The HDL taken from the homozygous CETP deficiency patients, however, failed to substitute lacking of HDL for embryonation of the eggs (Figure 5) (Okumura-Noji et al., 2001). Take-up of CE by the eggs was less from the HDL isolated from a patient with CETP deficiency (Figure 6). The findings were reproduced by using the plasma of mouse, where the wild type is a CETP deficiency model lacking plasma CETP activity and the CETP transgenic mouse is accordingly a model for normal human (Okumura-Noji et al., 2001). In the infection of *S. japonicum* in the mouse model, the number of the aberrant eggs in the liver was not different between the wild-type and CETP-transgenic mice, but the ectopic embryonation to miracidia was significantly more in the CETP-transgenic mice (Figure 7) (Okumura-Noji et al., 2001). Most interestingly, development of granulomatosis was found more also in the transgenic mice, indicating that lack of CETP leads to resistance to the fatal hepatic complication in Schistosomiasis by *S. japonicum* (Table 1) (Okumura-Noji et al., 2001).

**TABLE 1:** Granulomatosis lesions in the liver (Okumura-Noji et al., 2001).

	CETP transgenic	Wild type
Granuloma Area (%)	10.5 ± 5.5	14.4 ± 5.4
Area per egg (µm^2^) × 10^–3^	86.7 ± 26.7	66.1 ± 19.3*

## Endemic of CETP Deficiency

CETP is a plasma protein to catalyze equimolar non-directional exchange of CE and triglyceride (TG) among lipoproteins (Ohnishi et al., 1994a; Ohnishi et al., 1994b; Ko et al., 1994; Qiu et al., 2007) in certain species of mammalians including humans (Bruce et al., 1995; Jiang et al., 1995; Tall, 1995; Wang et al., 1995). Human CETP is composed of 476–amino acid residues (Drayna et al., 1987) with a glycosylated molecular weight of 74 kDa. The reaction equalizes distribution of the core lipids among lipoprotein particles and accordingly causes net transfer of CE from HDL to TG-rich lipoproteins such as VLDL and TG reversely to HDL and LDL (Ko et al., 1994). CE is generated on HDL in plasma by enzymatic cholesterol acyl-esterification and TG in VLDL and chylomicron originates in the liver and the intestine, and CETP reaction moves CE out of HDL and, in turn, transfers TG both to HDL and LDL where TG is hydrolyzed by hepatic or lipoprotein lipases. Accordingly, the size of HDL and LDL becomes smaller to produce HDL3 and “small dense” LDL. The increase in plasma TG, in fact, decreases plasma HDL and increases small dense LDL in the presence of CETP, and HDL-CE increases by decrease of CETP reaction (Föger et al., 1996).

Generation of CE in HDL is one of the driving forces to remove cell cholesterol (Czarnecka and Yokoyama, 1993) to play one of the key roles in cholesterol transport from peripheral cells to the liver for its catabolism. CETP reaction facilitates this pathway by transferring HDL-CE to LDL for its efficient recovery by the hepatic LDL receptor (Francis et al., 1991). On the other hand, increase of HDL surface by decrease of CETP activity may provide more capacity to accept cell cholesterol.

The patients with CETP deficiency were first described in Japan in 1985 as the cases with hyperalphalipoproteinemia (Koizumi et al., 1985; Kurasawa et al., 1985). Its genetic background was soon established (Brown et al., 1989), and many cases were found in Japan thereafter. Two major genotypes have been identified as intron 14 G (+1)-to-A (Int14A) and exon 15 missense mutation (D442G) (Inazu et al., 1994; Sakai et al., 1995; Hirano et al., 1997). Prevalence of these two mutants was found very high among Japanese general population as 1%–2% and 6%–7%, respectively. Many sporadic cases were also identified with more than 10 other types of mutations among Japanese (Yamashita et al., 2000; Maruyama et al., 2004; Nagano et al., 2004). The heterozygotes can therefore be estimated around 10 million, and the homozygotes would be as many as 150,000 to 250,000 in Japan. CETP deficiency may account for 27.6% of the people with HDL cholesterol ≥60 mg/dl and 31.4%–32.5% of those with HDL ≥80 mg/dl in Japan (Inazu et al., 1994; Nagano et al., 2002). City of Omagari, Akita district in Northern Japan, was found with high accumulation of the Int14A mutant, showing the prevalence of the heterozygote 27% (Hirano et al., 1997). Thus, genetic CETP deficiency is highly common among Japanese.

The first non-Japanese patient was found in Switzerland as a Chinese descendant (Ritsch et al., 1997). Several reports thereafter described CETP deficiency among Asians. Prevalence of D442G mutant heterozygotes was found as 3.3%**–**10.8% among the mainland Chinese (Chiba et al., 1997; Hui, 1997; Chen et al., 2008) and 4.5%**–**7.7% in the population of Taiwan (Hsu et al., 2002; Jap et al., 2002; Nagano et al., 2002; Yang et al., 2006). It could be estimated as 12% among Koreans based on its allele frequency 6% (Song et al., 1997). Vietnamese D442G mutants were estimated as 6.9% in their general population (Thu et al., 2005). Nine cases were found as D442G heterozygotes among the 35 hyperalphalipoproteinemia individuals in Thailand accounting for 26% (Plengpanich et al., 2009) same as Japanese (Inazu et al., 1994; Nagano et al., 2002). Detailed information should be referred to the previous review article (Thompson et al., 2009). Interestingly, analysis of elderly Siberian Yakuts showed the prevalence of D442G mutant 16.2% in the native Yakuts and 5.2% among the non-indigenous (Arkhipova et al., 2013), mostly Russians and Ukrainians whose intermarriage with Yakuts is 10%–20% (Arkhipova et al., 2013; Yokoyama et al., 2015). No reliable information is available for Int14A mutation, except for two out of the 145 subjects (1.4%) in Hong Kong Chinese (Ma et al., 2001; Okumura-Noji et al., 2001; Takahashi et al., 2001; Zhang et al., 2001; Hsu et al., 2002; Nihei et al., 2006; Finkelstein et al., 2008) and none in the 346 Vietnamese (Thu et al., 2005). CETP deficiency is thus highly prevalent widely in East Asia, predominantly with D442G mutant. Int14A may be second common but it is unclear except in Japan. Many other types of mutations have been found in Japan but information from other Asian regions is not adequate. The data are summarized in Table 2.

**TABLE 2:** Frequency of CETP deficiency in various regions of the world as prevalence of mutants in population (%).

	D442G	In14	Number Genotyped	References
Japanese Americans	5.1	0.49	3,469	Zhong et al. (1996)
Japanese medication free	8.1	0.60	2,267	Arai et al. (2005)
Japanese controls (Osaka)	6.0	1.00	514	Hirano et al. (1997)
Japanese children	6.0	0.00	500	Arashiro et al. (2001)
Japanese on hemodialysis	6.5	—	414	Kimura et al. (1999)
Japanese FH	3.5	0.69	288	Haraki et al. (1997)
Japanese centenarians	6.3	0.78	256	Arai et al. (2003)
Japanese controls	6.8	1.69	236	Inazu et al. (1994)
Japanese high HDL	28	13	270	Nagano et al. (2002)
Japanese high HDL	13.7	4.42	226	Akita et al. (1994)
Japanese controls	6.8	1.58	190	Arai et al. (2003)
Japanese controls (Omagari)	4.0	27.0	173	Hirano et al. (1997)
Chinese controls	4.2	—	379	Hui, (1997)
Chinese controls	5.0	1.00	335	Zhuang et al. (2002)
Chinese controls	3.3	—	209	Zheng et al. (2004)
Chinese CHD	10.8	—	203	Zheng et al. (2004)
Chinese CHD	3.5	0.00	200	Hui, (1997)
Chinese stroke	3.6	0.91	110	Zhuang et al. (2002)
Chinese Healthy elderly	3.0	—	103	Xia et al. (2005)
Chinese MI	3.5	1.05	94	Zhuang et al. (2002)
Hong Kong Chinese	—	1.4	145	Ma et al. (2001)
Taiwan Chinese controls	6.7	—	718	Hsu et al. (2002)
Taiwan Chinese controls	4.7	—	278	Wu et al. (2001)
Taiwan Chinese controls	4.5	—	224	Jap et al. (2002)
Taiwan Chinese CHD	7.7	—	196	Wu et al. (2001)
Korean	11.3	—	270	Song et al. (1997)
Korean postmenopausal	9.2	—	228	Han et al. (2002)
Vietnamese	6.9	0.00	348	Thu et al. (2005)
Thai high HDL	26		35	Plengpanich et al. (2009)
Yakuts	16.3	—	144	Arkhipova et al. (2013)
Siberian Russians/Ukrainians*	5.2	—	116	Arkhipova et al. (2013)
North Indian controls	0.0	0.0	315	Dixit and Mittal, (2005)
French healthy controls	0.0		100	Xia et al. (2005)
Scottish case/controls	0.0		1,606	Freeman et al. (2003)
Caucasians**	(<1.0)			Thompson et al. (2009)

*According to personal communication with the authors, 10%–20% are of cross-marriage with Yakuts. **Description without reference in (Thompson et al., 2009).

In contrast, CETP deficiency seems rare in any other ethnic groups. The first Caucasian case was reported in 1997 in North America (Teh et al., 1998), and one case with Int14A was reported in 1998 in Canada without ethnic identification (Hill et al., 1997). It was thus said that CETP deficiency is rare among North American Caucasians (van der Steeg et al., 2007). A few studies later added sporadic cases of CETP deficiency in the United States (Rhyne et al., 2006), Italy (Calabresi et al., 2009; Cefalù et al., 2009), and the Netherlands (van der Steeg et al., 2007; Chantepie et al., 2012). There was an estimation of D442G mutant among Caucasians “less than 1%” without evident grounds (Thompson et al., 2008) (Table 2).

The only significant clinical manifestation in CETP deficiency is abnormal profile of plasma lipoprotein spectrum characterized by very high HDL cholesterol and moderate reduction of LDL cholesterol. Lack of CETP reaction accumulates CE in HDL particle to expand the core to make it as large as LDL and rich in apoE (Inazu et al., 1990; Yamashita et al., 1990; Sakai et al., 1991; Yamashita et al., 1991) and causes heterogeneity in size and lipid composition of LDL due to lack of core lipid equilibration (Yamashita et al., 1988; Hirano et al., 1992). The patients do not suffer from any serious clinical symptoms in general. Roles of CETP deficiency in atherogenesis are controversial as potential increase of the risk in the homozygotes (Hirano et al., 1997), whereas overall risk may decrease among the heterozygotes (Moriyama et al., 1998; Goto et al., 2001; Curb et al., 2004). The outcome of its pharmacological inhibition has also been inconclusive so far (Nissen et al., 2007; Schwartz et al., 2012; Bowman et al., 2017).

## Is *S. japonicum* Associated With High Prevalence of CETP Deficiency in East Asia?

Two potential mechanisms are considered for geographic or ethnic accumulation of a genetic abnormality: “founders’ effect” and screening by a regional fatal disease(s), which are mostly infectious in these situations. The former cases are found in relatively isolated communities of the descendants from earlier settlers. Typical examples are familial hypercholesterolemia accumulated in French Canadians (Bétard et al., 1992), in Afrikaners of South Africa (Torrington et al., 1984) and maybe in Lebanese (Abifadel et al., 2009). In this case, the accumulated mutations are not highly diverse as originating in a few carrier families. On the other hand, the latter is represented by sickle cell anemia and other hemoglobinopathies that is resistant to malaria infection and therefore stated to be a reason for its high prevalence among African ethnic groups (Allison, 1954; Hedrick, 2012; Taylor et al., 2012). This case may affect larger populations historically exposed to specific diseases. There are more examples to implicate similar association (Withrock et al., 2015) such as cholera and cystic fibrosis (Muanprasat and Chatsudthipong, 2013), *tuberculosis* and Tay–Sachs disease (Spyropoulos et al., 1981), mycotic abortions, and phenylketonuria (Woolf et al., 1975) likely causing selective pressure. The others are also suggested resistance but not to a selection level such as enveloped viruses and disorders of glycosylation (Sadat et al., 2014), filoviruses and Niemann–Pick C1 disease (Carette et al., 2011), and rabies and myasthenia gravis (Lafon, 2005).

Majority of the patients with CETP deficiency accumulated in East Asia may be limited to one or two type(s) of mutations but further diversity of mutation is also found in the region. In addition, the region with high prevalence of the mutants is so large in East Asia beyond considering founder’s effect. No specific settler family can be conceivable to account for such large descending population affected. The only exception is extreme accumulation of the mutant int14A in Omagari, Japan, which can be a potential case of the local “founders’ effect” (Hirano et al., 1997). However, the only significant phenotype of CETP deficiency is abnormal plasma lipoprotein profile to which very few infectious diseases are found related. Schistosomiasis is one of the few to meet such criteria as discussed above.

The reason for poor reactivity of the HDL of CETP deficiency is not clear at this moment. CETP is a plasma protein that mediates nondirectional transfer of hydrophobic core lipids like CE and TG between lipoprotein particles (Ohnishi et al., 1994b). As CE is enzymatically generated constantly in HDL particles in plasma, net transfer of CE to LDL and VLDL occurs mainly in exchange with TG by the reaction of CETP (Ko et al., 1994). In the lack of CETP, CE remains and accumulates in HDL to make it enlarged, sometimes as large as LDL as described above (Inazu et al., 2008). Such a large HDL may be dysfunctional in its cellular interaction and selective uptake of CE for some reason. Remodeling of HDL by CETP was shown to result in enhancement of CE uptake from HDL by SR-B1 in cultured cells (Collet et al., 1999) and expression of CETP in mice enhanced HDL-CE uptake in the liver (Harada et al., 2007). These findings are consistent with the observations that CD36-RP is not functional to take up CE from the HDL of CETP-deficient plasma.

**FIGURE 8:**
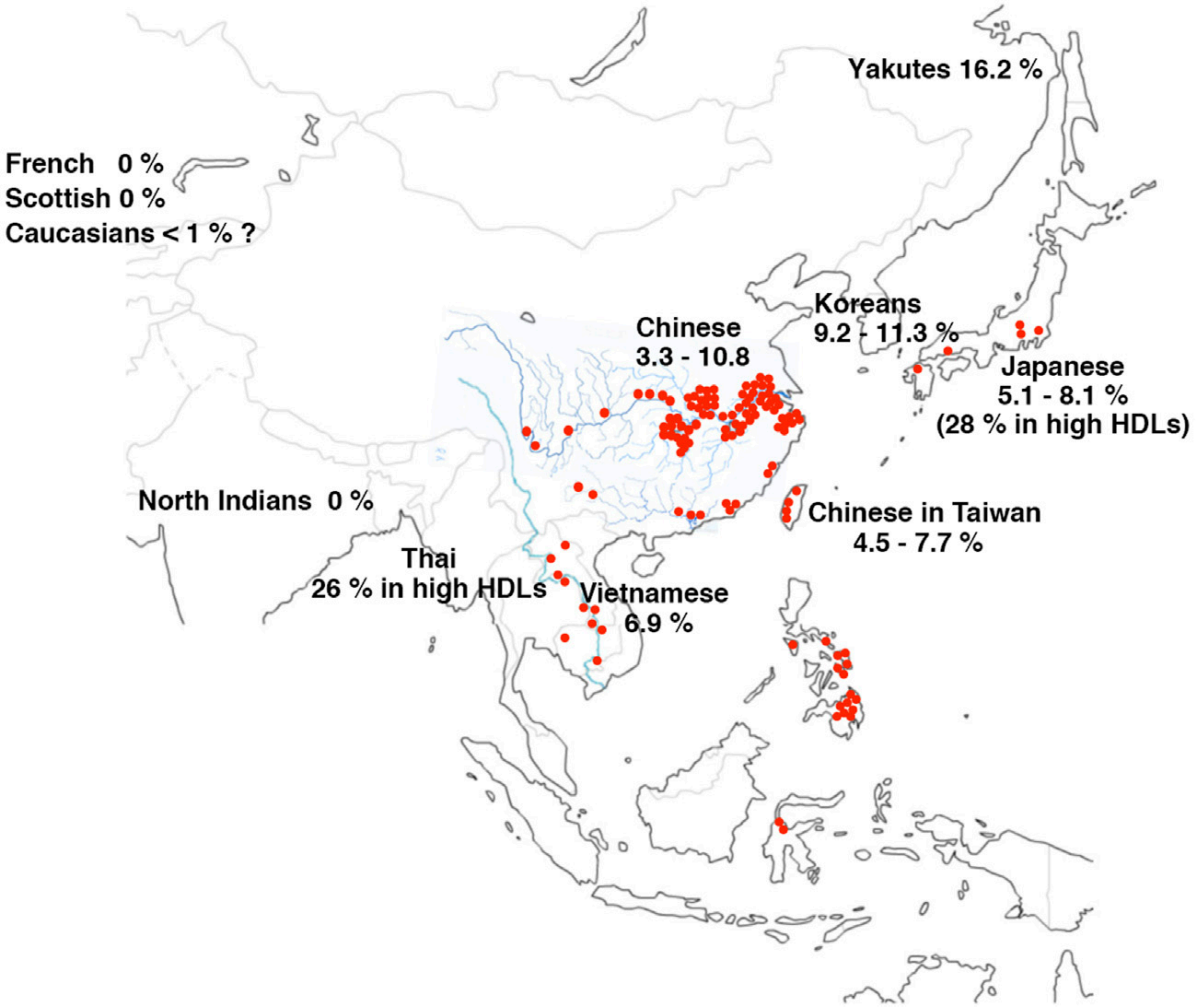
Endemics map of *S. japonicum* and CETP deficiency (D442G). Red spots show the regions where the cases of infection by *S. japonicum* were found currently and historically since the early 20th century when the parasite was identified. The regions spread over Japan, China including Taiwan, Mekong valleys, Thailand, Philippines, and Indonesia. The endemic was eliminated in many of these regions by the 21st century. Data of the prevalence of G442G available in literature till today are listed as percentage in each ethnic general population as listed in Table 2.

The regions the mutations are found with high prevalence of CETP deficiency largely overlap with the areas where *S. japonicum* infection is either currently still endemic or it has been or potentially been highly endemic in the historic records (Figure 8; Table 2) (Yokoyama, 2014; Yokoyama, 2015; Yokoyama et al., 2015). The high prevalence among Yakuts may indicate genetic drift from their Asian ancestors. Many of these areas have been civilized under the culture of water farming of crops such as rice, so that their common life style fits for incubation of Schistosome life cycle. As the homozygotes of CETP deficiency are apparently resistant for development of the fatal complication of *Schistosomiasis japonica*, i.e., hepatic granulomatosis and cirrhosis in the mouse model (Okumura-Noji et al., 2001), it is conceivable that this disease was one of the factors that caused high prevalence of the CETP gene mutations in the East Asia.

## Conclusion

*S. japonicum* expresses CD36RP in the adults and their eggs, which mediates selective CE uptake from the host plasma HDL as a pathway for the parasite to ingest lipid nutrients (Okumura-Noji et al., 2013). This reaction seems essential for the parasite eggs to embryonate to miracidia, likely not only for generation of the yolk before they are laid but also for replenishment after being laid. Large abnormal HDL caused by CETP deficiency becomes a poor substrate for the CD36RP-mediated CE uptake (Okumura-Noji et al., 2001; Okumura-Noji et al., 2013), so that the aberrant eggs into the liver are to grow less to miracidia and generate less granulomatosis in the patients with CETP deficiency. Thus, *S. japonicum* infection can be one of the factors to screen CETP deficiency in East Asia. Because the ectopic embryonation is the major fatal complication of schistosomiasis, the CD36RP pathway is an important potential target to inhibit for prevention of the fatal complication of this disease (Okumura-Noji et al., 2013). Inhibition of CETP can be an approach to block this process and prevent the liver complication when the infection outbreak is feared such as a case of major flooding in the endemic region. Alternatively, CD36RP can be a novel vaccination target to prevent the liver complication. The data are still limited to *S. japonicum* so that *S. mansoni* should be investigated to find whether the findings are more general. Further studies are required to understand association of *S. japonicum* infection and CETP gene mutation based on genome-wide analysis for evolutional backgrounds of the human genes in certain regional or ethnic groups, like the recent findings of copy number variants in Melanesians selected from Denisovans and Neanderthals (Hsieh et al., 2019) or lack of inheritance from Neanderthals in East Asians of genetic risk factors for clinical severity of COVID-19 (Zeberg and Pääbo, 2020; Zeberg and Pääbo, 2021).
